# Digest: Recent movement of a flicker hybrid zone

**DOI:** 10.1111/evo.14571

**Published:** 2022-07-19

**Authors:** Jente Ottenburghs

**Affiliations:** ^1^ Wildlife Ecology and Conservation Wageningen University Wageningen The Netherlands; ^2^ Forest Ecology and Forest Management Wageningen University Wageningen The Netherlands

## Abstract

What biotic and abiotic factors drive hybrid zone movement? By resampling a classic hybrid zone between two subspecies of woodpeckers, Aguillon and Rohwer documented a westward shift that may be attributed to changes in land use or climate. These findings highlight the increasing influence of anthropogenic activities on hybridization events.

Hybrid zones—geographic areas where distinct populations meet and interbreed—are not static but rather dynamic phenomena. Indeed, the location of several hybrid zones has shifted over time (Wielstra 2019). The movement of hybrid zones can provide important insights into the fitness of the hybrids compared to their parental taxa. In a tension zone, for example, hybrid fitness is lower than parental fitness, resulting in a balance between selection against hybrids and dispersal of parental taxa into the hybrid zone. Tension zones might thus move depending on the dispersal rate of the parental taxa and the density of the hybrid population (Barton and Hewitt 1985). If, on the other hand, the fitness of hybrids is higher in particular habitats (i.e., a hybrid superiority zone; Moore 1977), the hybrid zone might move by tracking shifts in the suitable habitat where hybrids thrive. However, documenting hybrid zone movement is not an easy feat and often requires repeated sampling over time.

In this study, Aguillon and Rohwer (2022) revisited the classic hybrid zone between the yellow‐shafted flicker (*Colaptes auratus auratus*) and red‐shafted flicker (*c. a. cafer*) in the Great Plains of North America. Applying geographic cline analyses on several plumage traits of more than 350 individuals, they showed that this hybrid zone has moved ca. 73 km westward between historic (1955–1957) and more recent (2016–2018) times. Previous work indicated no reduction in intrinsic hybrid fitness within the hybrid zone (Moore and Price 1993), suggesting that the movement of the hybrid zone may instead be linked to environmental changes. A possible explanation involves land use changes that resulted in woody encroachment, providing the flickers with more breeding opportunities. Alternatively, climate change might have shifted the favorable habitat for hybrids westward.

Regardless of the exact mechanism, it seems likely that human‐induced changes have contributed to the movement of this hybrid zone. Several recent studies have already highlighted the influence of anthropogenic activities on hybridization. These studies mainly focused on the occurrence of novel species interactions and consequent hybridization, including the introduction of non‐native species and range shifts due to land use changes (Grabenstein and Taylor 2018; Ottenburghs 2021). The work of Aguillon and Rohwer (2022) shows that anthropogenic changes can also influence existing hybrid interactions. As humans continue to alter the environment and affect species distributions, more hybrid zones are expected to be influenced. Although these situations pose difficult challenges for the conservation of endangered species, they also lead to unique opportunities for evolutionary biologists to study the speciation process. It will, however, require the repeated sampling of classic hybrid zones.

Associate Editor: k. Moore

Handling Editor: T. Chapman

## SUBMIT A DIGEST

Digests are short (∼500 word), news articles about selected original research included in the journal, written by students or postdocs. These digests are published online and linked to their corresponding original research articles. For instructions on Digests preparation and submission, please visit the following link: https://sites.duke.edu/evodigests/.
